# Small footprint optoelectrodes using ring resonators for passive light localization

**DOI:** 10.1038/s41378-021-00263-0

**Published:** 2021-05-26

**Authors:** Vittorino Lanzio, Gregory Telian, Alexander Koshelev, Paolo Micheletti, Gianni Presti, Elisa D’Arpa, Paolo De Martino, Monica Lorenzon, Peter Denes, Melanie West, Simone Sassolini, Scott Dhuey, Hillel Adesnik, Stefano Cabrini

**Affiliations:** 1 The Molecular Foundry, Lawrence Berkeley National Laboratory, Berkeley, CA 94720 USA; 2 Department of Applied Science and Technology, Politecnico di Torino, Torino, 10129 Italy; 3 Adesnik Lab, University of California Berkeley, Berkeley, CA 94720 USA; 4 aBeam Technologies, Hayward, CA 94541 USA; 5 Lawrence Berkeley National Laboratory, (LBNL), Berkeley, CA 94720 USA

**Keywords:** Optics and photonics, Nanobiotechnology

## Abstract

The combination of electrophysiology and optogenetics enables the exploration of how the brain operates down to a single neuron and its network activity. Neural probes are in vivo invasive devices that integrate sensors and stimulation sites to record and manipulate neuronal activity with high spatiotemporal resolution. State-of-the-art probes are limited by tradeoffs involving their lateral dimension, number of sensors, and ability to access independent stimulation sites. Here, we realize a highly scalable probe that features three-dimensional integration of small-footprint arrays of sensors and nanophotonic circuits to scale the density of sensors per cross-section by one order of magnitude with respect to state-of-the-art devices. For the first time, we overcome the spatial limit of the nanophotonic circuit by coupling only one waveguide to numerous optical ring resonators as passive nanophotonic switches. With this strategy, we achieve accurate on-demand light localization while avoiding spatially demanding bundles of waveguides and demonstrate the feasibility with a proof-of-concept device and its scalability towards high-resolution and low-damage neural optoelectrodes.

## Introduction

Exploring the human brain has emerged within both academia and industry as a multidisciplinary challenge^1^ aimed at understanding how information is processed and results in mental functions and behavior^2^ as well as at gaining insight into diseases, such as Parkinson’s and other neurological disorders^3^.

Invasive in vivo devices such as Michigan probes^4^ integrate a variety of sensors and stimulation sites that locally record and manipulate neural activity with high spatial (few µm) and temporal (sub-ms) resolution^5,6^. Arrays of electrodes record the neuron extracellular potentials and enable the triangulation of the neuron positions by measuring differences in signal timing and amplitude^7^; however, neural stimulation with electrodes results in interference with the electrophysiological recordings and cannot target specific types of neurons^8^. Conversely, light stimulation through micro-light-emitting diodes (µLEDs)^9^ or small waveguides^10,11^ yields fast and cell-type-selective optogenetic manipulation of neural circuits^12,13^.

State-of-the-art probes integrate both arrays of electrodes and light sources to record neurons while optically stimulating them, implementing feedback loops with high spatiotemporal resolution^14–16^ Specifically, these probes aim at (i) recording signals from high numbers of neurons by integrating multiple sensors^15^, (ii) optically stimulating specific neural populations or groups by delivering light to the location(s) of interest in a (iii) passive fashion—meaning that no electrical currents that generate heat are required^10^—and (iv) reducing the implant size to minimize brain damage^17^.

Several technologies address one or more of these requirements. For example, probes with µLEDs and electrodes^9,18^ selectively stimulate specific cortical layers but at the cost of heat generation during µLED operation. Alternatively, combining µLEDs with waveguides enables passive and multiwavelength illumination^19,20^ but does not allow for spatially conveying light in different device areas^19^ or device miniaturization^20^. This is due to every light output corresponding to a different waveguide, resulting in bulky devices, limited numbers of sensors and stimulation sites, and high cross-sectional area coefficients (i.e., the ratio between the tip cross-section and the total number of sensors and stimulation sites)^20^.

Tapered optical fibers^21,22^ and nanophotonic circuits^12^ deliver light to the area(s) of interest but do not integrate electrodes. Recent solutions for integrating electrical switches and nanophotonic circuits have led to the stimulation of neurons with millisecond temporal resolution^23^ but lack the possibility of miniaturization. Thus, to the best of our knowledge, no device combining both electrodes and passive nanophotonic elements enables spatial control of the light emission location while providing a reduced footprint and a high sensor density.

**Fig. 1:**
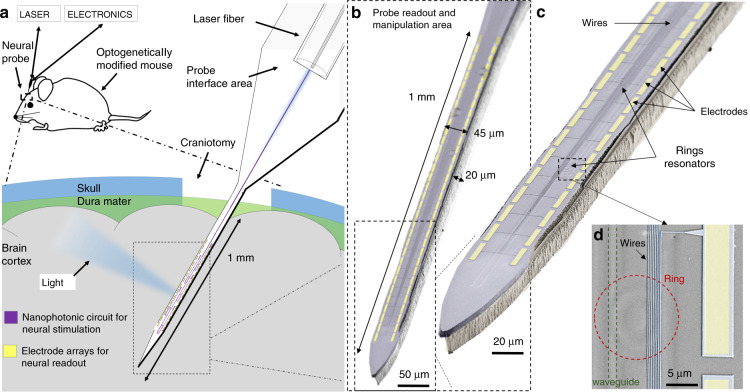
Small footprint and passive optoelectrode for electrophysiology and optogenetic applications. **a** Schematic illustration of the neural probe, which is a needle device inserted into the brain cortex of an optogenetically modified mouse to study neural functions. The probe tip, which is the only part of the device inserted into the cortex, has a minimally invasive size for reduced brain damage and integrates both nanophotonic circuits and arrays of sensors for simultaneous high-spatiotemporal-resolution optical manipulation and readout of neural networks. We connect the probe to an external laser and electronics through the device interface area to access the tip circuit functions. **b** False-color scanning electron microscopy (SEM) image of the probe tip showing the integration of both arrays of sensors (yellow) and nanophotonic circuits and highlighting the length (1 mm), width (45 µm), and thickness (20 µm) of the tip. The scale bar is 50 μm. **c** Close-up image of the probe tip, showing the wires and electrodes (highlighted in yellow) as well as the underlying nanophotonic circuit with ring resonators. The scale bar is 20 μm. **d** Further close-up showing wires and electrodes on top of some nanophotonic components: the bus waveguide (highlighted with dashed green lines) and a ring resonator (dashed red circle). The scale bar is 5 μm

To overcome this technological challenge, we integrate ring resonators^24^ into our neural probe along with a high number of sensors for simultaneous electrical readout (Fig. 1a–c). Rings are gaining interest as optical switches for wavelength division (de)multiplexing applications^25^ in various fields (biosensing^26^, lasing^27,28^ and computing^29,30^). Compared to other nanophotonic technologies (such as arrayed waveguide gratings or electro-optic modulators), rings combine high speed (<µs), low power consumption (fJ)^29^, and a small footprint with integrability into arrays of dozens of independently selectable channels^31^.

Here, we design nanophotonics with multiple rings to spatially address the light output location along the probe tip; small shifts of the external laser wavelength (<1 nm) lead to each ring being selected, preventing any electrical current that could generate heat. All of the rings are coupled to a single input waveguide, thus resulting in a small lateral footprint along the probe tip (<35 µm); above the nanophotonics and in a separate layer, we integrate a high number of sensors (64) while maintaining small tip dimensions (45 µm width, 20 µm thickness). The resulting device has a cross-sectional area coefficient of 12, which is one order of magnitude lower than that of state-of-the-art optoelectrical probes^20^.

To demonstrate the feasibility with a proof-of-concept device, we perform all the design, fabrication, and characterization stages, followed by preliminary in vivo testing. We show that our strategy, which integrates both arrays of sensors and nanophotonic circuits with embedded ring resonators, effectively combines various ideal features for optoelectrodes: implant size reduction, increases in the numbers of sensors and stimulation sites, and light localization without heat generation.

## Results

### Optoelectrode architecture

Our probes integrate both arrays of sensors for neural activity readout and nanophotonic circuits for passive and on-demand stimulation of the areas of interest. We combine micro- and nanofabrication techniques to optimize the device reproducibility and achieve high throughput and scalability. The probe (schematically illustrated in Fig. 1a) consists of three parts: (i) the tip, which is the electrical readout and stimulation area and the only part inserted into the mouse cortex; (ii) the interface area, which connects the tip circuits to the external laser and electronics; and (iii) a connecting area, which brings the electrical and optical signals from the tip to the interface area and vice versa.

Along the 1 mm long, 45 µm wide, and 20 µm thick tip (Fig. 1b–d), we integrate small footprint nanophotonic circuits and electrode arrays. The nanophotonic circuits, which we describe in detail in the next section, allow for choosing the light output location passively since switching is enabled through the wavelength sensitivity of the circuits and external wavelength control. As a result, no electrical currents flow through the optical elements, avoiding any heat generation other than that caused by light itself, which is negligible compared to electrical heating^32,33^.

Electrode arrays for neural activity recording are integrated into a separate layer above the nanophotonics to keep the tip width as narrow as possible while increasing the numbers of sensors and stimulation sites. We describe this layer thoroughly in the “Integration of electrode arrays” section.

Both circuits are connected to an external laser source as well as electronics through the probe interface area, which comprises a groove for an optical fiber and large electrodes for wire bonding. We report the fabrication and assembly steps in a dedicated section (“Device fabrication and assembly”).

### Nanophotonic circuits: design, and realization

Nanophotonic circuits allow for selective illumination of specific locations along the probe tip to stimulate neighboring neurons of interest. We select the desired light output by routing the light confined in the nanophotonic circuits by means of ring resonators^24^, which act as passive optical switches. Different resonators can be selected simply by tuning the laser input wavelength via minor wavelength shifts (<1 nm) without requiring an electrical current flowing through the optical elements. In addition, rings have the advantage of a small footprint, as only one main waveguide (also referred to as a bus waveguide) is needed to interface with all of the ring resonators, as opposed to other configurations that require one waveguide for each output spot.

**Fig. 2:**
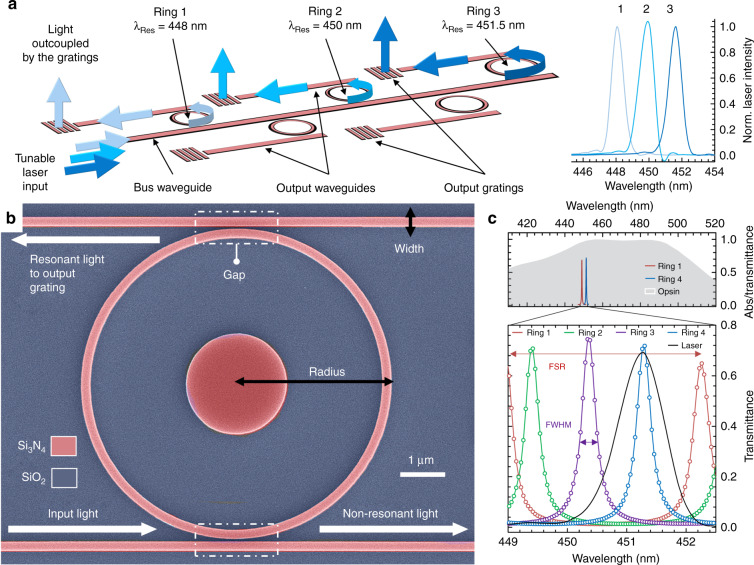
Small footprint nanophotonic circuit for passive light localization by the use of ring resonators. **a** Schematic illustration of the nanophotonic circuit in the probe tip area, comprising an input waveguide, ring resonators (with different radii and, therefore, different resonance wavelengths), and corresponding output waveguides and light extraction gratings. Arrows indicate the light path, with their color corresponding to the laser input spectrum, reported in the right panel. **b** False-color SEM image of a silicon nitride (Si_3_N_4_, colored in pink) ring resonator on top of a SiO_2_ substrate (gray area) with well-defined radius (3.881 μm), gap (80 nm), and width (250 nm). White arrows indicate the light propagation direction. The scale bar is 1 μm. **c** FDTD simulation of the ring transmittance (amount of light transferred from the bus to the output waveguide) for the implemented ring radii. Top panel: Ring 1 and 4 resonances (red and blue solid lines, respectively) reported together with the ChR2 opsin absorption spectrum (gray shading, from ref. ^37^). Bottom panel: magnification of the wavelength range between the ring 1 and 4 resonances (solid colored lines) in the top panel. FDTD simulated spectra for the four rings are displayed, together with the laser spectrum (solid black line) centered at 451.3 nm. The FSR is highlighted with a red arrow to show that it fits within the laser tunability range

The nanophotonic circuit (Fig. 2a) consists of a bus waveguide, several ring resonators (which we place along the length of the tip at an optimized distance from the bus), and, for each ring, an output waveguide terminated by a grating^34^. When the input laser light from the bus matches the ring resonance frequency, which, for a given material and thickness, is mainly a function of the radius, the light resonates due to constructive interference and transfers to the output waveguide, where it is extracted by the grating. Based on this model, we design rings with different radii such that they resonate with different input wavelengths in our range of interest, as shown in the right panel of Fig. 2a. With this strategy, we can passively select each ring and its relative light output location.

We set the initial wavelength range for the ring resonance frequencies according to the maximum absorbance of the channelrhodopsin ChR2 (centered at 450 nm^35^) and then restrict it according to the tunability range of our laser, 3.4 nm (laser model: QFLD-450-10S from QPhotonics). We then optimize the ring parameters (gap, width, and radii, shown in Fig. 2b) using finite difference time domain simulations (FDTD, Lumerical)^36^.

We choose a ring free spectral range (FSR), which is the wavelength spacing between two resonances pertaining to the same ring^24^, of 3.21 nm; this value is close to the laser tunability range to maximize the number of rings. The resulting ring activation wavelength range (449–452.5 nm) fits within the maximum CHR2 opsin absorption range; therefore, we do not expect a significant variation in the opsin sensitivity at the different grating sites^37,38^.

We then set the ring Q factor (~1600) to be on the same order of magnitude as that of the laser (860 ± 20) to maximize the percentage of light transferred from the bus to the output waveguide at the resonance frequency (called transmittance). Given the chosen FSR and Q factor, we design four ring resonators with an average ring transmittance of 69.5 ± 2.9%, as shown in Fig. 2c along with the opsin absorption (light gray area) and the laser spectrum (black curve).

The overlap between different ring resonances could result in light leaking into unselected rings. We calculate a 6% overlap between resonance curves, which results in an on/off ratio (the amount of grating output power for the selected ring versus that for the unselected ring) of 12.2 dB. This value is comparable with those of other nanophotonic technologies^23^, and it ensures a sufficient gap between the output power at the desired grating versus that at the unselected gratings. As a result, once the proper light output power range for the system under study is chosen, the leaking light will not activate unwanted neurons.

Furthermore, our system can be extended to dozens of individual channels by choosing a laser source with a broader tunability range^39^ and extending the ring FSR with series-coupled resonators^40^. Such an increase in the number of rings does not increase the tip lateral dimension due to the reduced nanophotonic element size and only one bus being required to interface all of the rings. We choose a distance between gratings for the current design of 150 µm; however, our strategy can accommodate much denser light output configurations depending on the design of interest (see the Methods Section). Overall, our design yields a nanophotonic circuit with a lateral footprint as low as <35 μm (or <15 μm by placing the rings on a single side of the bus waveguides), thus meeting the desired feature of a small tip size.

### Integration of electrode arrays

**Fig. 3:**
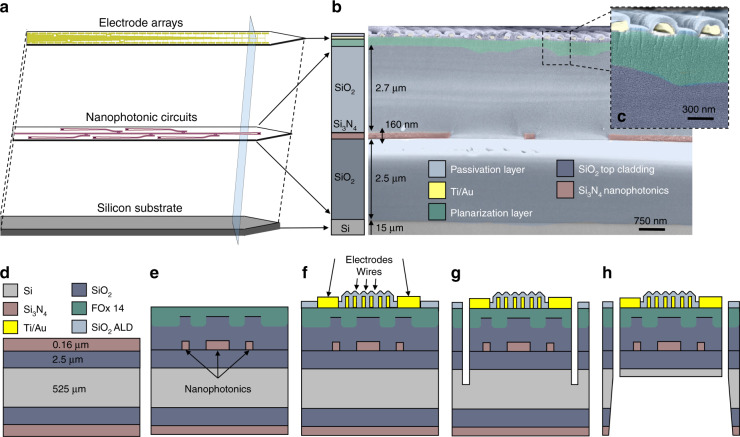
Schematics of the integrated device and its fabrication process. **a** Stacking of the nanophotonic circuits and electrode arrays. **b** False-color SEM cross-section, with the corresponding scheme on the left, highlighting the different layers. The scale bar is 750 nm. **c** High magnification of the top layers in (**b**), showing the planarization layer and wires. The scale bar is 300 nm. Fabrication steps: **d** Initial silicon wafer (525 µm), with 2.5 µm of LPCVD SiO_2_ and 160 nm optical grade Si_3_N_4_ layers. **e** Patterning of nanophotonic circuits, optical insulation, and planarization using a flowable oxide (FOx 14). **f** Integration of the arrays of electrodes and wires above the nanophotonic circuits, followed by passivation with 60 nm of ALD-grown SiO_2_. **g** Patterning of the device shape. **h** Release of the device by backside etching, which removes most of the underneath silicon and yields an overall probe thickness of 20 µm

Arrays of electrodes, which enable the readout of neural activity during light excitation, are integrated above the nanophotonic circuits (Fig. 3a) to maximize the numbers of sensors and stimulation sites in the given tip area. The integration of the arrays of electrodes must be defined on a planar surface to avoid wire collapse due to the severe roughness stemming from the presence of Si_3_N_4_ nanophotonic elements. Hence, we planarize the substrate with a 350 nm thick flowable oxide layer^41^, as shown in Fig. 3b, c.

The resulting readout circuit in the tip consists of 64 closely packed titanium/gold electrodes with lateral dimensions of 5 µm × 25 µm and a pitch of 27.5 µm and 64 corresponding electrodes in the probe interface area. Note that other electrode designs can be chosen according to the brain area of interest. We connect pairs of electrodes at the two ends of the device with lithographically defined metallic wires passivated by SiO_2_, which are 120 nm wide with a 450 nm separation between them in the tip area and widen to 1 µm in the interface area.

### Device fabrication and assembly

We fabricate nanophotonic circuits and arrays of sensors and integrate them on actual tips using micro- and nanofabrication techniques, allowing us to obtain ~200 devices per wafer.

The device fabrication process, sketched in Fig. 3d–h, starts from a commercial silicon wafer with SiO_2_ and Si_3_N_4_ optical quality layers^42^ (Fig. 3d). We initially pattern the nanophotonic circuits by electron beam lithography, followed by dry etching, cladding them with 2.7 µm of PECVD-deposited SiO_2_ (Fig. 3e) and spinning and baking of flowable oxide (FOx 14 from Dow Corning)^41^ to planarize the substrate. We then align and pattern the arrays of sensors with electron beam lithography, titanium/gold evaporation, and liftoff. We proceed with wire passivation by depositing 60 nm of SiO_2_ with an atomic layer deposition tool; other thicknesses or materials could be used, but based on the material dissolution rate (<1 nm/day^43^), we expect to be able to use the probes for several chronic studies. We then remove the electrode passivation layer through another electron beam lithography step and dry etching (Fig. 3f), thus leaving the SiO_2_ coating localized on the wires only. We choose electron beam lithography for convenience and design flexibility in the patterning of the nanophotonic circuits, arrays of sensors, and passivation layer opening; however, other lithography techniques could be used for batch fabrication and higher throughput.

Next, we pattern the profile of the devices and the grooves for alignment of the optical fiber from the wafer frontside (Fig. 3g) by using optical lithography and dry etching. We finally release the devices from the wafer by backside wet etching in potassium hydroxide solution (Fig. 3h), which removes most of the silicon underneath the tip area to achieve a 20 µm thickness while leaving the bulk silicon underneath the probe interface area. This device fabrication is fundamentally based on the processes described in further detail in ref. ^34^.

**Fig. 4:**
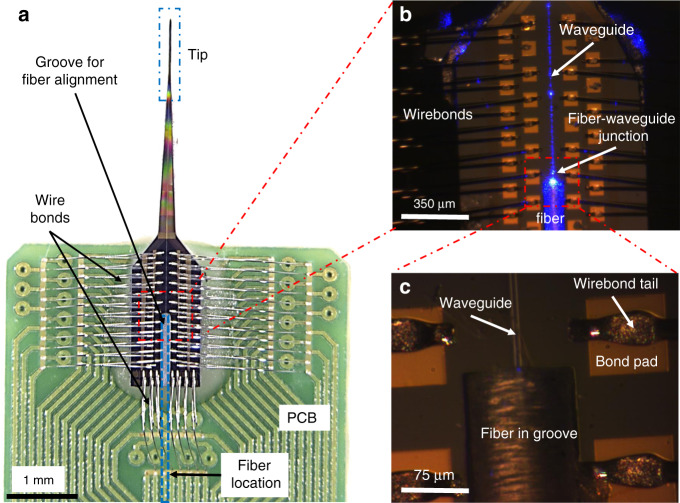
Optical microscope images of the probe after connecting its nanophotonic circuits and arrays of sensors to external instrumentation. **a** Device electrical connection. The whole device is shown, consisting of the tip and the interface area, which has bonding pads and an alignment groove for the optical fiber. The probe is glued onto a custom-made printed circuit board (PCB) and wire bonded. The scale bar is 1 mm. **b** Magnification of (**a**) showing the optical junction between the fiber and the waveguide, as well as the wire bonding and groove area. The laser is on, and the waveguide (in blue) is visible due to sidewall scattering. The scale bar is 350 µm. **c** Close-up image of the fiber-waveguide edge junction, highlighting details of the fiber, waveguide, and surrounding bonded pads. The scale bar is 75 µm

To access the tip circuits and use their relative functions, we connect them to external electrical instrumentation and a laser source. Specifically, we achieve electrical connection by gluing and wire bonding our probe on a custom-made printed circuit board (PCB, shown in Fig. 4a), which has electrodes on one side for wire bonding and Samtec electrical connectors on the other side. Moreover, we obtain optical connection by coupling the laser single-mode optical fiber to the edge of the bus waveguide (Fig. 4b, c). We maximize the alignment between the fiber and the waveguide by using piezo actuators while monitoring the probe output, then dispense a low shrinkage, UV curable glue, and cure it through the optical fiber with 405 nm wavelength light to fix the fiber to the sample and secure the alignment.

### Optical characterization of the probe

Once we fabricate and assemble the neural probe, we test its electrical and optical functionality in saline. The electrical characterization consists of measuring the electrode impedances and lowering them from 5.4 ± 0.43 MΩ to 0.202 ± 0.012 MΩ (at a 1 kHz frequency) through electrodeposition of metallic nanoparticles; details are discussed further in the “Materials and methods” section.

The optical characterization aims at validating the capability of the nanophotonic circuit to address the light output location and estimate the device output power and losses. In the following, we measure the ring coupling efficiency, the on/off ratio, and the output power of selected and unselected gratings.

**Fig. 5:**
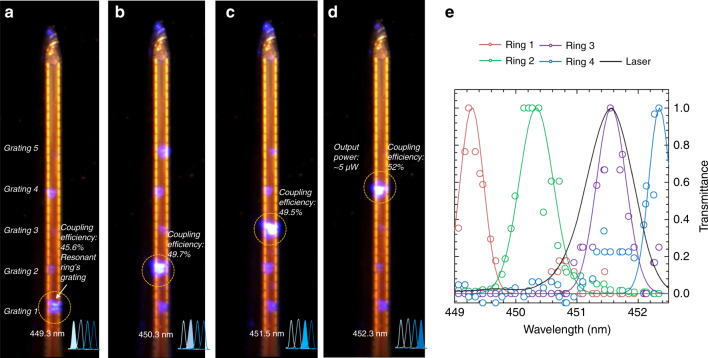
In vitro probe optical characterization. Optical microscope image of the tip of the assembled neural probe after turning on the laser at the first four ring resonance wavelengths: **a** 449.3 nm, **b** 450.3 nm, **c** 451.5 nm, and **d** 452.3 nm. The number of each grating is reported in (**a**) and is the same in (**b**–**d**). For each image, we report the resonant grating coupling efficiency. All the gratings in the image are visible through oversaturation for graphical purposes. The scale bar is 50 µm. **e** Normalized experimental ring transmittances for different ring resonators (colored circles, with the Gaussian fits reported as solid lines in corresponding colors) and laser spectrum (solid black line)

We connect the laser (model QFLD-450-10S, from QPhotonics; see more information in the “Materials and methods“ section) to the fiber we had previously aligned and glued to the nanophotonic circuit input. We then monitor the tip output gratings under an optical microscope while simultaneously tuning the laser wavelength (within a range of 3.4 nm). Moreover, we tune the laser polarization with a paddle controller to match the polarization that we designed for the ring resonators (TE fundamental mode). An example of this test is depicted in Fig. 5, where we show a tip with five ring resonators. We demonstrate light spatial localization after (Fig. 5a–d) we turn on the laser at the wavelengths corresponding to the first four ring resonances. A video of the light output switching is available in the Supplementary Information. We measure the ring output transmittances and plot them in Fig. 5e along with the laser spectrum. From this measurement, we extract the experimental ring Q factor (on average: 861 ± 127), which closely matches that of the laser (860 ± 20). The ring coupling efficiency, calculated as each grating output intensity divided by all the grating intensities, is between 45 and 60%. The experimental cross-talk is 5.2 ± 2.6%, which results in an on/off ratio of 13.4 ± 2.4 dB. This value is in excellent agreement with the simulated on/off ratio (12.2 dB).

The ring output intensities show good uniformity, with a standard deviation of 11.3%. Note that the ring free spectral range is not available since it is wider than the tunability range of our laser.

Finally, we evaluate the tip output power, which, for a given laser input power, is limited by the device losses (~30 dB) that are mainly ascribed to fiber-waveguide coupling (~15 dB) and waveguide scattering (~8 dB) (see details in the Methods Section). System loss minimization is crucial to output sufficient light power for activating the opsin, which was estimated to be ~1 mW/mm^2 ^^35^.

At the maximum output power of our laser of 10 mW, the power at the selected grating is ~5 to 10 µW. Given the grating dimensions of 5 µm × 10 µm, we estimate a power density of ~100 mW/mm^2^, which shows that our system outputs more power than necessary to activate the opsin.

Our system allows for choosing a proper range of output powers so that sufficient power will stimulate neurons at the desired grating, while the output power at unselected sites is more than one order of magnitude lower and therefore unable to activate neurons. For instance, when sending an output power density of 1 mW/mm^2^ to a grating, corresponding to the opsin activation threshold, only  ~50 µW/cm^2^ will leak into undesired rings, thus leaving the unselected neurons unaffected by residual light.

### In vitro and in vivo experiments

Next, we perform a preliminary in vivo test to demonstrate a proof of concept of the probe, which aims to verify that we can simultaneously read neural activity across the entire tip and optically stimulate neurons in selected vertical areas of interest. We select specific areas of interest by choosing specific output grating(s) and matching the input laser wavelength to the corresponding ring resonance. A mouse that had been previously implanted with a headplate and that showed good viral expression of the light-sensitive opsin ChR2 (more details in the Methods Section) is selected for the in vivo characterization experiment. Cells expressing ChR2 simultaneously express TdTomato, a red fluorophore, allowing us to easily target the region with the brightest expression as our recording area. We record signals from the vibrissae somatosensory cortex after we insert the probe through a small (<200 µm) craniotomy, lower it to 1000 µm into the brain, and allow the electrode to settle for 5 min before beginning our experiment.

Throughout the experiment, we record neural activity simultaneously across multiple electrodes, from which we identify and distinguish individual units (presumptive neurons) and estimate their vertical positions along the probe, as shown in ref. ^34^ (using data postprocessing algorithms; see the Methods Section).

Specifically, we divide the experiment into 14 blocks, each composed of 100 trials that are three seconds long, in which the laser is turned off for one second, on the next second, and then off for one second. We group the blocks into subgroups, each corresponding to fixed laser temperature/wavelength and to one of the output gratings. By selecting a fixed laser temperature and a short experimental duration (300 s), we ensure the wavelength stability of our laser (see the “Materials and methods” section). However, our nanophotonic design can be used for fast (ms) stimulation experiments since ring resonators operate at sub-µs timescales^29^. This requires the use of a more reliable and fast switching tunable laser (such as the laser described in ref. ^10^) to enable long-term recording and/or fast (ms) optogenetic stimulation.

**Fig. 6:**
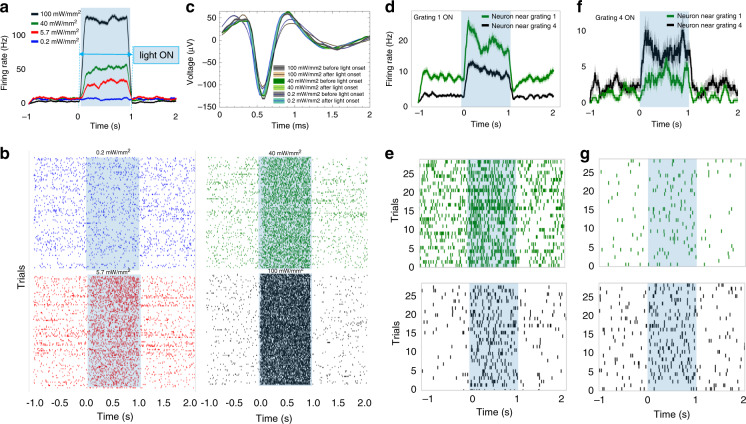
In vivo recording and simultaneous optogenetic excitation experiment performed in a mouse expressing the ChR2 opsin. **a** Firing rate of a neuron before, during, and after light excitation at different intensities, and **b** corresponding raster plots. The firing rate increases proportionally for intensities above ~5.7 mW/mm^2^ (*n* = 100 trials, mean ± sem). **c** Mean waveforms of the neuron of (**a**, **b**) before and after light onset for different grating output intensities, showing no changes in the waveform amplitude or shape. **d**–**f** Excitation of neurons in different spatial locations based on the activated light output grating. Firing rates of two units (**d**, **f**) and corresponding raster plots (**e**, **g**) when either grating 1 or grating 4 is on (*n* = 30 trials, mean ± sem)

We begin our experiments by sending light with increasing power. We choose a different input power for each block in a subgroup, spanning over 3 orders of magnitude. The grating output power changes accordingly, from 0.2 mW/mm^2^ to 100 mW/mm^2^. Within each subgroup, we observe that increasing the laser input power increases the neuron firing rates (number of action potentials over time, Fig. 6a, b). Importantly, we note that the firing rates increase when the light output of a specific grating is above a critical threshold. Specifically, we observe neural activity excitation for a laser input of 0.5 mW, corresponding to a grating output power of ~0.28 µW and a power density of ~5.7 mW/mm^2^.

We compare and report in Fig. 6c the mean waveforms of the same neuron of Fig. 6a, b before and after the laser pulse (1 s time) at each reported power density. The waveform amplitude does not change as a function of light power, as indicated by the small standard deviation; this shows that the detected neuron firing rate increases due to the optical stimulation of ChR2 rather than due to photoelectric artifacts.

Next, we investigate whether we can modulate the firing rates of the units by selecting different gratings. As shown in Fig. 6d–g, by selecting different optical gratings, we cause the firing rates of the units to change dramatically; the rates of the units increase or decrease, likely as a function of their relative spatial position around the probe. Specifically, in Fig. 6d, we report the firing rates of two neurons, one close to the activated grating (neuron near grating 1) and one positioned far from the grating (neuron near grating 4). The traces are calculated from the raster plots in Fig. 6e and show a stronger activation of the neuron close to the activated grating (grating 1). By comparison, the opposite effect is recorded when grating 4 is activated (Fig. 6f, g), in which the neuron close to grating 4 is activated more strongly than the neuron close to grating 1.

We note that some activation of neurons is indeed present near unselected gratings (Fig. 6d–f) as a consequence of power leakage at unselected sites. However, we would like to stress that we perform the measurements with an output power density of 100 mW/mm^2^ at the selected grating, which translates into ~5 mW/mm^2^ for nearby, unselected sites due to a cross-talk value of 5.2 ± 2.6% (on/off power ratio of 13.4 ± 2.4 dB). As reported in the literature, values of 1 mW/mm^2^ are high enough to excite opsin molecules. By lowering the output power by 1–2 orders of magnitude (down to a few mW/mm^2^), one can ensure that only the neurons close to the selected grating are activated, as a leaking power density of ~50 μW/mm^2^ is too low to be effectively absorbed by the opsin. Therefore, we will keep a lower laser power in future trials to reduce spurious activation of neurons near unselected gratings.

In addition, we are investigating multiple options for a more efficient design of the nanophotonic circuit: for instance, we could use a laser with a narrower linewidth and decrease the ring FWHM^44^, or conversely, we could couple a laser with a higher tunability range and space the ring resonances farther apart. Both strategies aim at reducing the ring curve overlap and cross-talk.

While these in vivo tests are only preliminary measurements, we confirm the proof of concept of the capability of the ring to activate neighboring neurons and, therefore, our strategy to achieve passive and selective neural activity stimulation with low footprint optoelectrodes.

## Discussion

Optogenetic techniques rely on the development of fast optical tools to stimulate neural activity with high spatial and temporal precision. One significant step in this direction is the design and realization of small footprint optoelectrodes that deliver light in the area(s) of interest.

State-of-the-art optoelectrodes integrate either micro-LEDs, which, however, generate undesired heat, or bundles of waveguides, which are spatially demanding, cannot be miniaturized and thus result in limited numbers of recording and stimulation sites.

In this work, we integrate ring resonators in a neural optoelectrode combining a high density of sensors and stimulation sites, high scalability, and the capability of addressing stimulation sites for on-demand manipulation of specific spatial regions without any significant heat generation. Moreover, we perform preliminary in vivo experiments in an optogenetically modified mouse and validate the proof of concept of the probe of simultaneous recording and locally stimulating spatial regions of interest in the brain.

Rings are optical components used for fast computing applications since they (de)multiplex optical signals in a fast (<µs) and wavelength-sensitive fashion; here, rings act as passive optical switches that can be selected by tuning an external laser wavelength to select the light location inside brain tissue. In addition to being passive and fast optical switches, rings have a small footprint and require a single input waveguide for multiple outputs, thus resulting in a nanophotonic circuit with a small lateral footprint (<35 µm) and enabling a substantial increase in the number of light output sites without increasing the tip lateral dimension. As an example, our optoelectrode has a cross-sectional area coefficient (the tip cross-section divided by the total number of sensors and stimulation sites^20^) of 12, which is one order of magnitude smaller than that of state-of-the-art optoelectrodes and confirms that we have overcome the stringent tradeoff between the number of outputs and the overall tip dimensions.

Our tip design can be implemented in a variety of optogenetic experiments, in addition to the application shown in this work, where we selectively stimulate groups of neurons (or cortical layers) while detecting signal propagation across the in vivo neural network. For example, our technology can stimulate cells in specific layers and monitor whether cells in different areas exhibit increased activity, thereby analyzing the functional connectivity between cortical layers. Our design also enables one to investigate the correlational structure of a neural network by computing the correlations between pairs of neurons across the electrode array (on either fast or slow time scales) and to observe how optogenetic perturbations alter the network structure. Finally, our optoelectrodes can be used in behaving animals to relate trial-by-trial changes in neural activity to changes in behavior.

In conclusion, our work demonstrates the feasibility of scaling down neural optoelectrodes by microfabricating highly integrated, scalable, and passively addressable neural probes that rely on a combination of arrays of sensors and nanophotonic circuits with embedded ring resonators. Our proof-of-concept device opens the path to numerous future directions, such as accessing wider opsin selection using additional wavelengths, further increasing the number of light output spots by extending the ring FSR, and enhancing the fabrication process by nanoimprinting, all with the final goal of exploring electrical signal propagation in the somatosensory area by selectively silencing or exciting individual cortical layers.

## Materials and methods

### Fabrication

We fabricate the neural probes using commercial silicon wafers provided by Lionix (525 µm thick) with low-pressure chemical vapor deposition of SiO_2_ (2.5 µm) and Si_3_N_4_ (160 nm); we perform all the fabrication processes at low temperatures (<400 °C).

Alignment marks are initially patterned onto the substrate with electron beam lithography (using PMMA C4 resist), followed by electron beam evaporation of titanium and gold (10 nm and 100 nm, respectively) and solvent liftoff (1 h in Remover PG at 80 °C).

The nanophotonic circuits and ring resonators are aligned to the marks and patterned with electron beam lithography (using ZEP 520A resist diluted at 50% and aquasave) and reactive ion etching (RIE, using CHF_3_/O_2_ chemistry with a 48:2 gas ratio and a forward RF power of 40 W). The process is repeatable across the wafer and tested for multiple wafers, with minimal variations in the waveguide width such that there is negligible change in the experimental performance.

The nanophotonic circuits are optically insulated by depositing 2.7 µm of SiO_2_ (with a plasma-enhanced chemical vapor deposition tool using 50 sccm 1%SiH_4_:Ar, 720 sccm N_2_O and 160 sccm N_2_ at 150 °C) and planarized by means of a flowable oxide (FOx 15 by Dow Corning, spun at 2000 rpm and baked on a hotplate at 350 °C for 45 min).

The arrays of sensors are patterned with electron beam lithography (using 100% ZEP 520A), titanium/gold evaporation and liftoff (as for the patterning of the alignment marks). To passivate the wires, 60 nm thick SiO_2_ is deposited with an atomic layer deposition tool (using a plasma and a temperature of 40 °C) and then selectively removed from the electrodes with another electron beam exposure and RIE etching. We detail these processes in ref. ^34^.

Trenches are then etched to define the probe shape as well as the grooves for the optical fibers. To do this, we spin an optical lithography resist to mask the probe regions (AZ 40XT-11D, spun at 1750 rpm for a 40 µm thickness). We use an Oxford Plasma lab 100 Viper tool to etch both the layers above the silicon (using 35 sccm CF_4_, 15 sccm Ar and 10 sccm O_2_, 150 W RF, 400 W very high-frequency power and a 20 °C table temperature) and 15 µm of silicon (using 90 sccm C_4_F_8_ and 60 sccm SF_6_ at 15 °C, 35 W RF, and 300 W VHF).

The silicon underneath the tip areas is then removed to make them thin (20 µm), while leaving the silicon underneath the probe interface areas. To achieve this, we etch the silicon nitride on the wafer backside with optical lithography (using the resist MAP-1215) and RIE etching (same parameters as for the etching of the nanophotonic circuits). Both the MAP-1215 and AZ-40XT-11D resists are removed with a 30-min soak in AZ-400T.

Most of the silicon (480 µm) is removed from the previously nitride-etched areas on the wafer backside using potassium hydroxide (KOH) after coating a protective polymer on the circuits (Protek B3), as we describe in ref. ^34^. In addition, during the KOH etching, we protect the wafer circuits by placing the wafer on a wafer chuck (from AMMT). After the KOH etching, we remove the last few remaining µm of silicon with dry etching in the VIPER tool (using SF_6_ and O_2_ chemistry).

### Ring resonator design

We design ring resonators with a waveguide width of 250 nm and a ring-waveguide gap of 80 nm. The first ring has a radius of 3.881 µm; the following rings have radius increments of 12 nm.

The spacing between ring resonators depends on the illumination of interest and the laser model, whose tunability range and FWHM limit the number of independently addressable ring resonators. The minimum spacing between rings can be as low as ~10 µm if the output waveguides are perpendicular to the bus (simulations show no difference between this configuration and the one shown in Fig. 2 that has output waveguides parallel to the bus).

### Laser and optical fiber preparation

#### Laser

**Fig. 7:**
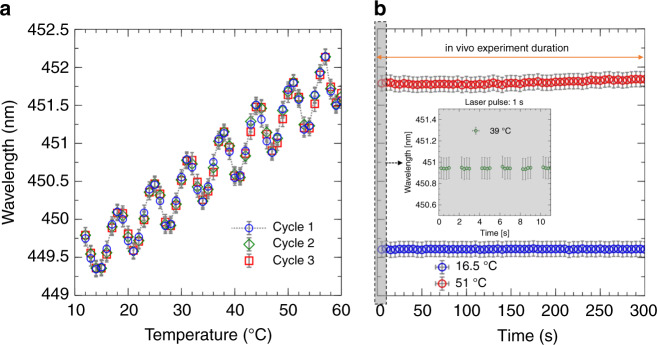
Laser diode temperature-wavelength calibration curve. **a** Output wavelength as a function of the temperature of the laser diode. Three different temperature cycles from 12 to 60 °C are reported (blue circles, green diamonds, and red squares correspond to the three cycles). **b** Output wavelength (same axis as in *a*) as a function of time over a 5 min interval, reported for two different temperatures, namely, 16.5 °C (blue circles) and 51 °C (red circles). The inset shows a similar recording of the output wavelength for a set temperature of 39 °C (green circles) during a 10 s time interval to show that the wavelength is stable over the 1 s period of the laser pulse during the experiment

We use a single-mode and fiber-coupled laser diode centered at a wavelength of 450 nm (model QFLD-450-10, from QPhotonics). We choose this small laser diode as a cost-effective proof-of-concept light source for testing our optical system. The diode has a maximum output power of ~10 mW. We tune the laser wavelength by changing the temperature through the laser controller in the range of 12–60 °C and monitor the corresponding wavelength with a spectrometer, as we describe in the following paragraph. We calibrate the diode to ensure reliable laser switching before conducting the in vivo experiments. As we cycle the laser temperature from 12 to 60 °C (with 1 °C increments and a 1 s relaxation time between each) and measure the same output wavelengths in every cycle, the laser repeatedly switches between different wavelengths, as shown in Fig. 7a. Once the laser temperature is set, the laser is stable since we do not measure wavelength shifts for 300-second-long time intervals, which correspond to the maximum duration of each in vivo experiment (Fig. 7b). In addition, we implement a closed loop that measures the wavelength with the spectrometer and automatically corrects the temperature in the case of wavelength shifts. Following these measurements, we can reliably use our laser model for 1-second-long pulsed stimulations with a fixed temperature for each experimental subgroup.

Different laser models with a wider tunability range, long-term stability, and fast switching can be fiber-coupled to our probes for future long-term recording and/or fast (ms) optogenetic stimulation.

#### Probe coupled fiber

We use single-mode optical fibers (model SM 400 from Thorlabs). We thin one side of the fiber such that it fits inside the 20 µm deep groove using either mechanical polishing or hydrofluoric acid etching. The first procedure is described in refs. ^45,46^ and allows for the mechanical removal of ~40 µm of the fiber cladding on one side. The second procedure is performed by immersion of the fiber in >40% HF for 45 min, followed by careful cleaning. This fiber is then spliced to a patch cord such that it can be readily connected to that of the laser.

### Ring resonator experimental measurement

We measure the spectral responses of the ring resonators by extracting their corresponding grating output intensities while changing the laser wavelength through its temperature control.

Since the wavelength of the laser is a nonlinear function of its temperature and its output power varies for different temperatures, we implement a feedback loop with a MATLAB script that adjusts both the temperature to match the desired wavelength (by tuning the temperature) and the laser output power to a constant value across every wavelength (by changing the input current).

### Device optical losses

The laser diode we use for the experiment has a maximum output power of ~10 mW. We estimate the total system losses to be ~30 dB. Of these losses, 1– 2 dB are due to the laser fiber-probe fiber FC/PC connection, 10–15 dB are due to edge coupling (10 dB) and fiber gluing (~5 dB), 6–8 dB are due to waveguide transmission losses (due to light scattering on the waveguide sidewalls), and 3 dB are due to grating outcoupling losses^30^. In addition, rings couple only 45–60% of the input light when their resonance frequency is matched (which introduces 3 dB additional losses). Therefore, the total output power is 5–10 µW, which corresponds to a power density of ~100 mW/mm^2^ under the assumption of a light output spot dimension on the order of the grating size (5 µm × 10 µm). The power is calculated from the microscope images by calibrating the CCD camera; we verify these calculations by comparing them with the probe output light measured with a power meter.

The system optical losses could be drastically reduced by further optimization: for example, propagation losses for nitride waveguides were reported to be below 1 dB/cm at a 532 nm wavelength^47^, and coupling and gluing losses could be reduced by using, e.g., grating coupling methods (~4.8 dB from our FDTD simulations) and by a more careful alignment (<3 dB), thus lowering the total losses from 30 dB to below 15 dB. Furthermore, most of the losses are at the fiber-waveguide interface in the probe interface area, which is far from the tip.

### Gratings

We simulate gratings with FDTD and use the following parameters: grating length and width of 15 ×4 µm, a pitch of 315 nm (for emission at 16° for a 450 nm wavelength), and a duty cycle of 0.5. Different grating designs could be chosen depending on the application of interest (e.g., focusing, as we described in ref. ^34^, or long-distance collimation^10^), but more detailed tests will be performed in future studies.

### Probe electrical characterization

**Fig. 8:**
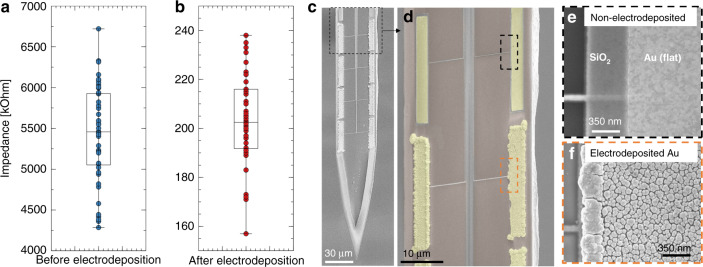
Probe electrical characterization. Electrode impedance measurement (**a**) before electroplating and (**b**) after electroplating gold nanoparticles. **c**–**f** SEM micrographs of the probe tip and magnifications of electroplated and nonelectroplated electrodes

We test the electrode impedance using the NanoZ toolkit. The average working electrode impedance at 1 kHz (Fig. 8a) is 5.4 ± 0.43 MΩ (nonworking electrodes have a > 10 MΩ impedance due to wire open circuits). We lower such high impedance values down to 0.202 ± 0.012 MΩ (Fig. 8b) by electroplating gold nanoparticles (Neuralynx) on the electrode surface at a −100 nA current for 6 s, which allows for increasing the surface area (Fig. 8c–f) and therefore enhances the system capacitance. Once we electrodeposit the nanoparticles onto the electrodes, we in vivo record single units, as shown in our previous work^34^.

### In vivo experiment

All experiments involving mice are performed in the Adesnik Lab, UC Berkeley, in accordance with the guidelines and regulations of the Animal Care and Use Committee (Protocol # AUP-2014-10-6832-1). The mice used in these experiments are wild type (CD-1, Charles River Laboratories) and undergo two surgical procedures in preparation for the in vivo optoelectrode tests. In the first procedure, the mice are anesthetized using 2% isoflurane and head-fixed to a stereotax using the proper aseptic technique. The scalp and the underlying fascia are removed to expose the dorsal part of the skull. Two small craniotomies are made using a dental drill, one over the vibrissae primary somatosensory cortex (vS1) and the other over the vibrissae motor cortex (vM1). A microinjector is used to inject 400 µL of an adeno-associated virus (AAV), carrying a genetic payload that causes infected neurons to produce Cre recombinase (Cre), into vS1. An additional injection of 400 µL of a second AAV, carrying a payload that causes neurons to express the excitatory ion channel channelrhodopsin (ChR2) in neurons that also contain Cre, is injected into vM1. These two brain regions share many reciprocal connections, giving us a large target area to test the probes. Once the injections are complete, the entire skull is covered in Vetbond (3M) to seal the wound margins and protect the skull. A custom aluminum headplate is attached to the skull using dental acrylic (Metabond). The mice are then taken off of isoflurane and allowed to recover. These mice are given a week to acclimatize to the new headplate and head-fixed to the rotary treadmill where the experiments occur.

The second procedure is conducted on the day of the experiment. Here, previously injected and headplate-attached mice that showed good expression of the excitatory opsin are anesthetized using 2% isoflurane. A small dental drill is used to thin the skull over the region of the brightest expression. After thinning, a 27 g needle is used to lift a small flap of the skull to expose the brain. Mice recover from anesthesia and are placed on the rotary treadmill in the electrophysiology rig. Here, the electrode is fastened to a micromanipulator (Sensapex) and lowered ~1000 μm into the brain, ensuring that all electrodes are inserted into the cortex. All neural recordings are conducted at a sampling rate of 30 kHz and recorded with SpikeGadgets hardware and software. During the recording, neural activity is clearly present and modulated with activation of the light pads on the probe.

Postprocessing of the data is conducted with custom MATLAB (Mathworks) and Python software. Semiautomated spike sorting is conducted using the open source software Klusta, which uses a custom sorting algorithm that takes the probe geometry into account to identify spike times associated with specific units.

## Supplementary information

Supplementary information video
